# Influences of peer facilitation in general practice – a qualitative study

**DOI:** 10.1186/s12875-018-0762-1

**Published:** 2018-05-28

**Authors:** Tina Drud Due, Marius Brostrøm Kousgaard, Frans Boch Waldorff, Thorkil Thorsen

**Affiliations:** 1 The Research Unit for General Practice and Section of General Practice, Department of Public Health, University of Copenhagen, Copenhagen, Denmark; 2 Research Unit for General Practice, Institute of Public Health, University of Southern Denmark, Odense, Denmark

**Keywords:** Facilitation, Facilitators, Outreach visits, Primary care, Qualitative study, General practice

## Abstract

**Background:**

Practice facilitation is increasingly used to support guideline implementation and practice development in primary care and there is a need to explore how this implementation approach works in real-life settings.

We focus on a facilitation intervention from the perspective of the visited practices to gain a more detailed understanding of how peer facilitation influenced practices and how they valued the facilitation.

**Methods:**

The facilitation intervention was conducted in general practice in the Capital Region of Denmark with the purpose of supporting the implementation of chronic disease management programmes. We carried out a qualitative study, where we observed 30 facilitation visits in 13 practice settings and interviewed the visited practices after their first and last visits. We then performed a thematic analysis.

**Results:**

Most of the respondents reported that facilitation visits had increased their knowledge and skills as well as their motivation and confidence to change. These positive influences were ascribed to a) the facilitation approach b) the credibility and know-how associated with the facilitators’ being peers c) the recurring visits providing protected time and invoking a sense of commitment. Despite these positive influences, both the facilitation and the change process were impeded by several challenges, e.g. competing priorities, heavy workload, problems with information technology and in some cases inadequate facilitation.

**Conclusion:**

Practice facilitation is a multifaceted, interactive approach that may affect participants in several ways. It is important to attune the expectations of all the involved actors through elaborate discussions of needs, capabilities, wishes, and approaches, and to adapt facilitation interventions according to an analysis of influential contextual conditions and change opportunities.

## Background

Various strategies are used to support guideline implementation and practice development in primary care, e.g. regulations, financial incentives, and information dissemination. A more active and increasingly widespread strategy is practice facilitation [1–8]. This is a multifaceted intervention, where an external person (most often a health care professional) visits the practice and supports a process of change [1, 7]. A systematic review and meta-analysis concluded that practice facilitation has “a moderately robust effect on evidence-based guideline adoption within primary care” [1]. However, there is considerable heterogeneity between the included studies and generally, there is no clear and consistent operational definition of facilitation. Hence, specific facilitation interventions vary considerably in their form and content. The literature portrays facilitators as having multiple roles and performing multiple activities [3, 6, 8, 9]. Among these are audit and feedback, consensus building, plan-do-study-act circles, provision of advice and education, cross-pollination of good ideas and support of internal discussions, and critical reflection. Recent contributions have emphasised the importance of tailoring facilitation to the specific needs and circumstances of the targeted practices [1, 10, 11].

Given the increasing popularity of facilitation, the flexibility of the concept, and the heterogeneity among interventions labelled as facilitation, there is a need to explore how facilitation is actually performed in real-life settings, how it affects practices, and how participants experience it. From January 2011 to December 2012, the Capital Region of Denmark carried out a facilitation intervention to support the implementation of chronic disease management programmes for type-2-diabetes and chronic obstructive pulmonary disease (COPD) in general practice. The intervention relied on general practitioners (GPs) as facilitators. In two previous studies, we explored how facilitation was enacted in this intervention and the effectiveness of the intervention [12, 13]. First, based on observations and interviews with facilitators we found that facilitation was enacted through four major roles: the teacher (knowledge dissemination), the super user (hands-on knowledge dissemination on the practice’s computer system), the peer (facilitators conveying their experiences and information about their own practice organisation), and the process manager (selection of topics, tasks, and status reporting at subsequent visits). We also found that the facilitators rarely enacted a more coaching based approach to encourage internal reflection and discussion during the visits [13]. Second, our randomised controlled trial on the intervention’s effectiveness showed mixed results. There was no difference between the allocation groups for the primary outcome (change in the number of annual chronic disease check-ups), but differences in some of the secondary outcomes (a higher reported use of ICPC diagnosis coding for type 2 diabetes, stratification for COPD and a faster initial sign-up rate for the Data Capture Module - a software program for patient overview) [12]. With the present study, we supplement our previous results by focusing on facilitation from the recipients’ (i.e. general practice) perspective to gain a more detailed understanding of how peer facilitation influenced practices and how they valued the facilitation. We also identify several factors, which inhibited the facilitation process.

## Methods

### Setting and intervention

The Danish health care system is primarily tax financed and offers free-of-charge access to general practice and public hospital services. The GP serves as the primary care provider and gatekeeper for patients’ referral to specialists and hospitals. They are private entrepreneurs, but mainly financed through the tax financed health care reimbursement scheme. The service provision of general practice is regulated via the collective agreement between the Danish Regions and the Organisation of General Practitioners [14, 15].

Chronic disease management programmes based on the Chronic Care Model [16, 17] have been developed in all five regions of Denmark [18]. The programmes outline evidence based treatment and a systematic approach to chronic care with division of tasks between GPs, hospitals and municipalities. They describe the GP’s role as coordinator of care and outline a systematic proactive approach with population based patient registration, annual chronic disease check-ups, and stratification of patients into three levels by risk of complications and complexity and state of the disease [19, 20].

Diverse initiatives have been initiated to support the implementation of the chronic disease management programmes and to improve chronic care management. The facilitation intervention in this study was one of these initiatives and it was developed and implemented by the Capital Region of Denmark. The overall aim of the intervention was to support the implementation of chronic disease management programmes for type-2-diabetes and COPD in general practice. Fourteen GPs were hired as facilitators. These differed concerning age, gender and practice type. They all went through an educational programme focused on the content of the disease management programmes and related tools, and on how to be a facilitator. All practices in the region were offered up to three visits of 1 h each. Visits were free of charge and the practices were compensated for lost income. The central principle of the intervention was that the practices’ own interests and choice of topics should drive the change process and that the facilitators therefore should tailor their activities to address the particular situation and needs of each practice. Thus, the intervention relied on the idea of a continuum of facilitator roles. The information material sent to the practices suggested relevant themes for the visits such as workflow procedures and division of tasks for chronic disease management, leadership and organisation, collaboration with municipalities and hospitals, the role of the GP as coordinator of care, and IT solutions for improved overview and systematisation, primarily the Data Capture Module (DCM). The DCM was a software program which automatically collected patient data from the GPs’ electronic health record system and provided individual and population based patient overview and data for quality improvement [21]. Shortly after the initiation of the facilitation intervention, sign-up to the DCM became mandatory and all practices were required to sign up no later than the 1st of April 2013.

The intervention has been described in more detail elsewhere [13]. As researchers, our role was to study the intervention and we were not involved in either the design or the implementation of the intervention.

### Methods

We chose an explorative approach for both data collection and analysis. Practices were strategically sampled [22], to ensure variation in geography, size, current level of development in areas relevant to the disease management programmes (assessed by initial questionnaires), and the associated facilitator. We observed 30 facilitation visits in 13 practice settings. Extensive notes were written and the visits were audio recorded. Further, the first author conducted group interviews in the 13 practice settings after their first and their last visit (4 of the 13 facilitator visits were joint visits where collaborating practices where present; hence, a total of 18 practices were represented). The group interviews lasted approximately 1 h, and we strived to include all GPs and staff who had been present at the facilitation visits. Table 1 presents an overview of the data material. As shown, the data collection was not complete in all practice settings. We audio recorded the interviews, transcribed them verbatim, and analysed them using thematic analysis [23]. We used the software program NVivo in the coding and theme constructing process for the interviews. We grouped codes in themes and sub-themes and then related the themes to each other and to the entire data material, thus refining and connecting them. The observations were primarily used to qualify the interview guides, but from the observation notes and audio recordings we also obtained information about task completion, the process between the visits, and potential challenges.

**Table 1:**
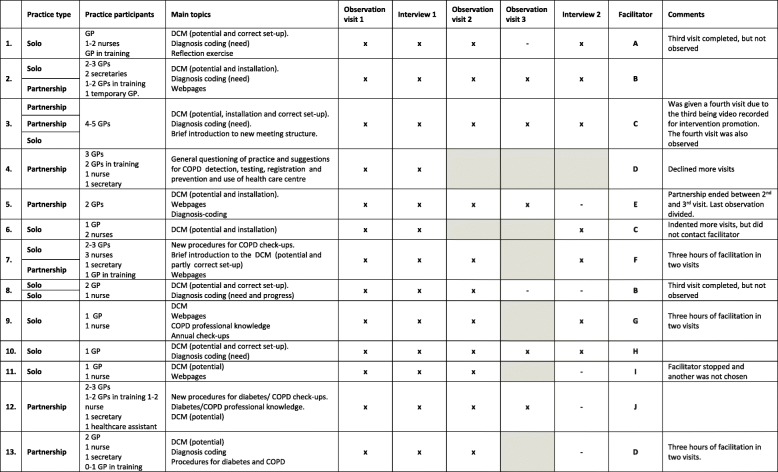
Participating practices and data material

“X” = Observed visits. “-” = Not observed visits or not interviewed. Shaded areas are not conducted visit

## Results

Prior to the visits, most practices only had a vague notion of what to expect from facilitation, and their understanding of the intervention was generally limited. Also the practices did not appear to experience a strong need for change. The dominant reasons for participating in the intervention was to get help with the DCM (because it became mandatory), or because the visits were seen as an occasion to get started with developing more systematic procedures for chronic care check-ups. A few practices had merely signed-up because a colleague had mentioned the intervention. Most of the observed practices chose the DCM as their main topic while two practices focused mainly on developing new chronic care procedures for diabetes and COPD (i.e. written descriptions of the workflow in the practice for a given disease, e.g. division of labour between GPs and nurses and amount and content of systematic check-ups). The topics of the visits are described in Table 1.

At the first visits, the practices decided on the topics of the visits. However, there was no introductory dialogue about the practices’ expectations or preferred facilitation approach and a limited clarification of their existing level of knowledge within the chosen topic. During the visits, the facilitators mainly engaged in various forms of knowledge dissemination, practical support and process management. Although the intervention design also comprised a more coaching based approach to support internal discussions and reflections (e.g. about existing and future procedures) this approach was not enacted during the observed visits. Still, the majority of the respondents were pleased with the visits and did not wish for this sort of facilitation approach. Several respondents appreciated the knowledge and inspiration offered by the facilitators, and some did not envisage that there was sufficient time at the visits for more elaborate discussions about their practice organisation. Nevertheless, two practices were quite dissatisfied with the visits because they had mainly expected the facilitators to engage the participants in an inspirational discussion about what changes were needed and how to implement them. Instead, they experienced the facilitators taking an educative stance which did not involve asking the participants reflective questions and which lacked a focus on implementation:

*it is not what a facilitator is supposed to do. When [the facilitator] is sitting on the side-line if you [the practice] are sitting and talking in the group, it is primarily making sure you do not lose focus, but also providing ideas in the process, saying… So that was what I had expected more of, more on the side-line, and then that we as a practice had tried to talk about how we would organise this. (GP, Practice 12)*

One of these practices described that they rarely set time aside for discussions about practice development. Therefore, they had hoped that the visits would have focused more on supporting their internal discussions and development processes, but they related that if a temporary doctor in training had not single-handedly taken upon her the task of making new procedures, they would not have accomplished much. In the other practice, the GPs were so disappointed with the facilitation style (being too educative and not enabling internal discussions) that they declined more visits.

Across the observed practices, profound changes in direct patient care were generally not initialised after the facilitation visits, but there were several examples of practices having initiated changes in some areas. Several practices increased their use of diagnosis coding and some installed and signed-up for the DCM, corrected the system set-up, and improved their data registration. However, none came as far as using the DCM data for quality improvement. Two practices formulated new chronic care procedures, and one of them had begun to implement it after the last visit. Additionally, a few practices expressed increased attention towards some of the addressed issues, e.g. annual chronic disease check-ups and the webpage for municipal chronic care activities. However, some practices did not express any tangible changes and some reported limited or no impact from the visits.

### Knowledge and skills

The facilitators provided factual knowledge about International Classification of Primary Care (ICPC) diagnosis coding of individual consultations in the electronic patient records, the content of chronic disease check-ups, the DCM, and websites on professional guidelines and municipal chronic care services to which GPs can refer patients. This was either done by presentations, by showing demo versions of the DCM, by demonstrating relevant websites, or by hands-on guidance in the practices’ electronic patient record systems [9]. Prior to the visits, most practices had not used the DCM. Some had not yet installed it and some had not managed to set up the programme to generate accurate data. Further, they rarely diagnosis-coded individual consultations and they had little knowledge (and made little use of) the various websites introduced by the facilitators. On this background, the practices experienced that the facilitation visits increased their knowledge and awareness both of new tools and how to use them, and of errors in the set-up of the DCM. Some respondents stated that the knowledge provided by the facilitators ensured a faster implementation process due to knowledge being more easily accessible, and others perceived the knowledge, especially about the correct set-up of the DCM, as being essential for progress, because they would not have figured it out themselves:

*We found out that we did not do it, that the computer was not set up properly... it turned out that the nurses’ computer was not set up to register the diagnosis-coding, which we had done through half a year. (Nurse, Practice 7)*

Respondents generally described the content of the visits as relevant, because they had chosen the topics themselves, and because these topics were closely related to their daily practice and specific challenges (experienced prior to and in-between visits). The respondents also found that conducting the facilitation meetings in the practice constituted a beneficial frame for knowledge provision. Contrary to lectures in larger settings, the facilitation visits focused on them, there were no disturbing questions from other practices, and they felt safe asking questions and revealing their weak points. Likewise, some appreciated that joint meetings in the practice increased the likelihood of the knowledge being applied, and relieved the GPs from spending time conveying it to the staff. However, other GPs preferred meetings without the staff so that the meetings focused on the needs of the GPs.

Regarding patient related data for quality improvement, the practices generally did not review their own data prior to or in between the visits. However, practices that looked at such data *during* the visits valued this experience. For them, the facilitation visits improved their appreciation of the relevance of patient data, helped them to identify problems, gave them an opportunity to consider data (which they could not usually find time for), and reinforced them to improve the registrations even more. A few practices also improved their skills in using their information systems due to the hands-on approach. The practices were generally satisfied with the technical knowledge of the facilitator. Nevertheless, some facilitators lacked knowledge about the specific IT systems used by the practice (there are 11 IT systems in Danish general practice), and several times they asked practices to contact their IT-providers with questions and problems they could not handle themselves. Some practices would have preferred a facilitator that had experience with their specific IT system, while others did not perceive this as a barrier. Several practices experienced IT challenges such as limited user-friendliness, errors in setting-up the DCM, and insufficient support from their IT system providers between the visits. This seemed to slow down the implementation process as some practices did not complete tasks or did so at a slower pace.

At the first visit, the facilitators did not clarify exactly what the visited practices wanted to focus on within a given topic or the level of their existing knowledge. Thus, although most practices reported that they obtained new knowledge from the facilitation visits, some of the knowledge provided was not new to everyone in the practices. While the GPs had generally gained little new knowledge from the presentations on medical and organisational aspects of chronic care, the practice staff often found this knowledge more relevant; not because it directly affected their own work, but because it improved their understanding of the GPs’ work. There were also several examples of participants forgetting the knowledge provided during the visits and several participants still had questions about the correct use of the DCM after the last visit. Some felt that too little time had been spent on some of the topics, that the visits had not been sufficiently structured and requested more written material on both the DCM and the facilitators’ organisation:

*One might have been given a sort of a template. Because the problem is that you forget it a bit afterwards…what is it you need to remember to implement it… perhaps one might have needed that. So a small action card. How to do it… because we cannot remember it now, right. (GP, Practice 13)*

### Motivation and confidence to change

According to most respondents the facilitation visits increased their motivation and confidence to change. They experienced the process of change as demystified and more manageable because the facilitators showed that the DCM was easier to use than they had assumed, and the facilitators’ descriptions of their own chronic care procedures gave them something to build upon:

*It might seem a bit less unmanageable and hopefully a little less time consuming than I feared it would be. (GP, Practice 5)*

*And*

*It was really good to get it [description of facilitators’ chronic care procedures], so you did not have to reinvent the wheel. (Nurse, Practice 7)*

Further, the facilitators’ descriptions of the benefits they had gained from making the changes in their own practices as well as the content of their chronic care procedures inspired the practices by increasing their sense of the changes being usefulness in daily practice. Most GPs found that it added to the credibility of the facilitators that they were peers with personal experience and knowledge of life in general practice. This meant that the GPs generally perceived the facilitators’ statements as relevant, trustworthy, and transferable to their own practice:

*I think it is true that a general practitioner will reach us more easily. We listen because there is a professional respect ... We listen more sharply and take it more seriously … than if it was a nurse… she would initially have to struggle against whether we could use it for anything. (GP, Practice 2)*

The GPs did not perceive the descriptions of the facilitators’ own practice organisation as something to be directly copied, but as a credible source of inspiration. The practices generally did not experience disadvantages from the facilitators being peers. Some could not see how the facilitators could have other professional backgrounds, while a few did not regard the peer component as crucial for the process. However, one of the previously mentioned dissatisfied practices felt provoked when the facilitator presented them with factual and experience-based knowledge because they did not perceive the facilitator as an expert or someone with an outstanding practice but just as a random GP. Also, while most GPs were motivated by the visits, some still expressed a feeling of obligation toward the DCM and doubted whether they would use the system beyond the required registrations:

*Well, it is the obligation that does it, because it is something that we have to do. If we had not had to, the question is whether we would have done it. That I don’t know. (GP, practice 3)*

Additionally, the technical problems experienced in the process triggered increased frustration with the DCM:

*Well it is just difficult to mobilise any energy among the doctors, who are to sit and code, if the shit does not work, excuse my directness. Then I bloody do not want to, and again I swear. Then I do not want to sit there and spend my time on something like that. Then it must be left to its own device until it is working. (GP, Practice 1)*

### Internal conditions for change

Three aspects of the intervention, which did not relate to the specific content of the visits nor to the specific skills and actions of the facilitator, influenced the change process and how the practices assessed the intervention.

First, the visits offered an occasion to focus on and initiate changes and provided protected time for this, which was much valued by the respondents, who reported on busy workdays where time was usually not set aside for practice development meetings with both GPs and staff attending. Thus, the visits were described as a timeout for development that accentuated the focus on the chosen topics:

*It also just helps quite a lot by creating a focus, because we devote an hour to it and sit here all of us together. Instead of in our busy workdays, where we just quickly went in and looked, and had set aside half an hour and then were fifteen minutes late and just got to look at something. Then this gives it much focus. (GP in training, Practice 2)*

However, sometimes the observed visits were delayed and sometimes people were absent or left during the meeting. Thus, while most respondents – for practical reasons – appreciated having the facilitator meetings in the clinic, some mentioned that this also increased the risk of interruptions and delays since patients were waiting before, during, or after the visits.

Second, practices reported that the visits supported task definition and delegation and increased the sense of obligation, agreement, and mutual responsibility because the whole practice attended the visits. However, from the observations it was clear that the clarity and systematisation of task definition and delegation varied and occasionally clear tasks were not explicitly defined.

Third, several practices described how the return of the facilitator at subsequent visits came to function as a reminder and deadline during the process. According to some respondents this speeded up the change process and ensured the completion of initiated projects that otherwise might not have been prioritised in a busy working day:

*So you knew, that you had a meeting at this and that date and suddenly, you were a bit more motivated to go in and code and do things…. So the meetings have another function than just being a meeting, they also have the function of keeping you up to scratch. (GP, practice 3)*

Thus, several practices managed to perform their delegated tasks and/or to set a deadline for their implementation before the next visit. Still, most practices rarely discussed the tasks or changes in the time between the visits and they explained this limited attention to the change process by referring to the daily time pressure in general practice.

## Discussion

Most of the respondents from general practice reported that facilitation visits had increased their knowledge and skills in relevant areas as well as their awareness of the need for change and their belief that change was possible and manageable. They also described having carried out tasks that otherwise would not have been completed, and they pointed to various features of the intervention that helped to generate these influences. Nevertheless, the impact of the facilitation visits mostly concerned intentions to change (or initial changes) rather than actual changes in chronic care management, and the study identified several factors which impeded the change process. Below we discuss these results using the theoretical model of behavior change proposed by Michie et al. [24], the COM-B model. According to the COM-B model, the three critical prerequisites for behavior change are: *Capability* (knowledge and skills required for change), *Opportunity* (enabling environmental resources), and *Motivation* [24]. Applying the COM-B model to our results, the various enablers and inhibitors of change in the facilitation intervention and its context may be characterized and understood as follows:

1. *Motivation:* The visits generally increased the participants’ desire and confidence to make changes. Most of the GPs found it important that the facilitators were peers, because this helped to establish the credibility of the facilitators and to increase the GPs’ perceptions of manageability and usefulness. This resembles the value that has often been ascribed to opinion leaders as change agents [25]. In both cases, much of the influence of the change agent is linked to the legitimacy and credibility gained by having worked under similar conditions. The participants’ motivation to change was also augmented by the recurrent visits which served as deadlines for the completion of the agreed-upon tasks. This sometimes appeared to be more influential for generating engagement and commitment than the specific content of the facilitation visits. Further, the DCM being mandatory often motivated the practices to focus on this change area. However, the motivation of some participants was negatively affected during the process due to the technical problems with the DCM. Further, the practices had diverse understandings and expectations in relation to the facilitation visits, and in some cases where these expectations were not fulfilled, motivation dropped.
2. *Capability:* The capabilities of the participants improved when the facilitators addressed the experienced challenges of the participants and engaged in a hands-on approach to knowledge dissemination. The facilitators also helped some participants to focus their change efforts and define specific tasks in practices with limited traditions for engaging in structured improvement processes. Yet, in some cases tasks were not made specific enough to promote change, and further some of the knowledge provided by the facilitators was redundant, inadequate, or forgotten. This latter problem with insufficient tailoring (which also concerned the motivational dimension, cf. above) suggests that a more thorough dialogue about current knowledge and preferred facilitation approach should have been initiated as the first step in the process. Such an approach could have optimized the perceived relevance of the knowledge provision and the style of facilitation and thereby increased the impact of the visits on both capability and motivation. A study by Watkins et al. likewise discussed the importance of an introductory talk about objectives and rules of engagement [26].
3. *Opportunity:* The opportunities of the participants for discussing and engaging in change were to some extent enhanced by the formal frames of the intervention providing protected time at three recurring visits. Still, the intervention did not provide additional resources (time or money) for the change process in between visits where most of the work was supposed to take place. This lack of influence of the participants’ opportunities for change seem critical for understanding the limited amount of actual changes in chronic care management generated by the intervention. Thus, contextual conditions inhibited the opportunities of the participants in several ways: First, the visits were sometimes delayed or interrupted due to urgencies in the clinic; second, the technical problems with the DCM wasted precious time; and third, some practices found it difficult to prioritize change efforts in between visits due to busy work schedules.

Previous studies have found that GPs appreciate facilitation visits for some of the same reasons as identified in this study, i.e. due to the contributions of the facilitators (motivating; giving advice and guidance in relation to specific problems; and helping with data mining and data correction) as well as the facilitation frames (offering protected time from the demands of daily work life and supporting a focus on change through recurrent visits) [6, 27–31]. Meanwhile several of the points mentioned above illustrate how contextual conditions may affect a facilitation process negatively, and similar impeding conditions (competing priorities, heavy workload and problems with information technology) have been identified in other studies [6, 28, 32, 33]. Since facilitation interventions always support change within a given context it is important to consider how the context will enable or weaken the capabilities, motivations and opportunities of the participants and how to deal with these influences when preparing the intervention – either by attempting to affect the context or by providing additional support as part of the intervention.

Berta et al. [34] have argued that the promise of facilitation lies in its potential to stimulate higher-order learning in organizations, and not just in supporting *single-loop learning* defined as corrective actions that “focus exclusively on improving efficiency of existing routines or processes” [34]. In contrast, *double-loop learning* occurs when organizations question the “initial goals, assumptions, and values that led to a particular workplace process” and this type of learning may “manifest as significant adaptive changes to workplace behaviours and routines and to goals, assumptions, and underlying values”. Furthermore, *triple-loop learning* is reflective “learning about learning” where learners “focus on learning that improves their learning processes, in addition to adaptive learning that improves production processes and optimizes behaviours” [34]. However, in our study it appeared that the learning which emerged from the facilitation visits only corresponded to single loop learning. Thus, the participants mostly focused on concrete practical changes and they did not seem to obtain tools to ensure future improved learning processes, or to challenge their existing values, objectives, or ways of working. While it is possible that a greater ‘dose’ of the coaching approach (encouraging practices to engage in more internal reflections and discussions of current practices) delivered over an extended period of time might have generated the kinds of higher order learning described by Bertha et al., this cannot be determined on the basis of our data. Nonetheless, the study also demonstrated that support on the level of single-loop learning was crucial for the improvement process in the practices, and hence the importance of single-loop learning should also be considered in future interventions.

### Strengths and limitations

Using interviews with participants as well as observations of facilitation visits is a strength of this study. Although rarely used in facilitation studies, observations provide a more nuanced picture of the facilitation process when combined with the practices’ reported experiences. Thus, the observations made it possible to explore less idealized versions of the facilitation process and to pose more nuanced and critical questions to the practices. It is also a strength that data was collected prospectively while the intervention was carried out since this reduced recollection bias among the participants and made it possible to explore the entire process. Potential limitations are that not all practices were interviewed twice and that the group interviews (where GPs and staff were interviewed together) might have inclined staff not to state conflicting opinions and made GPs more careful about criticizing the peer facilitator. Still, we deemed it important to give room for dialogue about a common experience between the various participants. Using qualitative methods, we generated detailed knowledge on how practices can be influenced by facilitation, connecting intervention activities and their impact, and uncovering types of influences, e.g. the sense of deadline, which likely would not have been identified with other methods. On the other hand, we do not know to what extend these findings apply to all the practices in the intervention, and the described impacts are not quantitative or standardized and therefore less comparable. However, a quantitative RCT based assessment of the intervention impact is previously reported [12].

Regarding the transferability of the findings beyond the specific setting both the enactment and the impact of facilitation are dependent on the content of the intervention as well as the institutional context and the the facilitators’ skills and professional background. Since facilitation interventions vary in their purpose and content and since they always take place in a specific context this put certain limits on transferability. However, as described in the discussion, some of the ways that the practices were affected by facilitation in this study and some of the influential contextual conditions have also been identified in other studies and should therefore be considered in future facilitation projects.

## Conclusion

In this study of practice facilitation in a real-life setting, most of the participants from general practice experienced that facilitation had increased their knowledge in some areas of chronic care and changed their perceptions of the relevance and manageability of making changes in these areas. Several elements of the intervention influenced the process positively such as the flexibility of the intervention (allowing participants to choose among several different topics), the provision of protected meeting time, the legitimacy and know-how of the peer facilitators, the focus on defining and delegating tasks, and the commitment associated with the deadlines set by recurrent visits. Despite the overall positive assessments of the participants, a number of internal and external factors impeded the facilitation process. Some of these challenges may be alleviated by a thorough initial discussion of the needs, capabilities, and wishes of the involved practices; by employing facilitators with diverse skills so that the different needs and starting points may be optimally matched by the individual facilitators; and by adapting the intervention according to an analysis of influential contextual conditions and change opportunities.

## Abbreviations

COPD: Chronic obstructive pulmonary disease
DCM: Data capture module
GP: General practitioner
ICPC: International classification of primary care
